# Maternal Impressions

**Published:** 1881-10

**Authors:** 


### SELECTIONS.

### MATERNAL IMPRESSIONS.

*Ed. Med. and Sur. Rep*:—

Will you allow me to indulge in a few remarks on "Maternal Impressions?" About thirteen years ago I attended a Mrs. H., in her first labor. Delivered the child with the forceps. No questions asked by the mother. The child had only one eye. Eyelids with lashes on right side were formed, but smaller than those of the left side, and no eyeball. I asked the mother whether she knew a cause for the deficiency, and she could at the time recollect none. She wondered whether I had not destroyed the eye with the instruments! I told her that such was not the case, since no wound could be shown to prove such a result. On my visit next day, she remembered that when she had passed her third month of pregnancy, she had an intolerable itching of her right eye; that she often stood before the looking glass and rubbed the eye with the knuckle of her index finger. The child's eye had the appearance of suspended development, occurring about the fourth month of its fœtal existence. Mrs. H's second child was born with red hair, fair skin, afterwards covered with freckles. Both parents have dark complexions and dark hair, and to the best of their knowledge, no red hair among either of their relatives. During her second pregnancy, on a certain Sunday, she stood behind her future brother-in-law, who had a head full of fine, fiery red hair, and admired its beauty. When her child was born it had a head full of fine, fiery red hair. Another child, born a year and a half later, by the same parents, also had fiery red hair. The first child, and three others, born subsequently, at intervals of two years, all have black hair and dark complexions. In their other features the children all resemble each other, excepting the hair, complexion and freckles.

Prof. Dunglison, during his lectures delivered at the Jefferson Medical College, Philadelphia, mentioned the instance of a male zebra and a female horse meeting at a certain watering place, the mare being at the time in heat. Connection followed, and the offspring, when born, had all the stripes of the zebra. The following spring the same mare was served by a male horse of her own color, and the offspring as in the first instance, had the stripes of the zebra. Prof. Dunglison ascribed the zebra stripes on the horse colt to the impression made on the female ovaries during her first meeting of the zebra. Be this as it may, I feel convinced that these occurences are not accidental, but incidental to a certain law of nature.

